# Nonmelanoma skin cancer in the Federal State of Saarland, Germany, 1995–1999

**DOI:** 10.1038/sj.bjc.6601294

**Published:** 2003-09-30

**Authors:** A Stang, C Stegmaier, K-H Jöckel

**Affiliations:** 1 Epidemiology Unit, Institute for Medical Informatics, Biometry and Epidemiology, University Hospital of Essen, Hufelandstr. 55, 45122 Essen, Germany; 2 Saarland Cancer Registry, Saabrücken, Germany

**Keywords:** epidemiology, skin neoplasms, incidence, registries, Germany

## Abstract

We analysed incidence data of basal cell carcinoma (BCC) and squamous cell carcinoma (SCC) of the skin from the Cancer Registry Saarland, Germany. During 1995–1999, the age-standardised incidence rates (world standard population) of BCC and SCC were 43.7 and 11.2 per 100 000 among men and 31.7 and 4.4 per 100 000 among women.

Nonmelanoma skin cancer (NMSC) is one of the most common malignant neoplasms in Caucasian populations around the world, and usually refers to either basal cell carcinoma (BCC) or squamous cell carcinoma (SCC) (Weinstock, 1994). Epidemiologic studies of these tumours have been limited by the fact that most patients are customarily seen and treated in the offices of physicians and not hospitalised (Scotto *et al*, 1983). Since the primary source of data for cancer registries is the in-patient hospital file, routinely collected statistics on NMSC are usually incomplete and not comparable with other forms of cancer (Scotto *et al*, 1996; Lucke *et al*, 1997). In addition, many cancer registries do not collect information on BCC and the incidence is often determined on the basis of surveys (e.g. Fears and Scotto, 1982; Giles *et al*, 1988; Green and Battistutta, 1990; Kricker *et al*, 1990; Roberts, 1990; Serrano *et al*, 1991; Green *et al*, 1996). In a recent review on the epidemiology of NMSC incidence, Green concluded that ‘little is known about worldwide trends in incidence of BCC and SCC’ (Green, 1992).

Here, we present the findings of detailed analyses of the NMSC incidence of the Federal State of Saarland for the period 1995–1999. We also report the incidence by histological groups and anatomical sites.

After the introduction of the International Classification of Disease for Oncology (ICD-O, first edition, WHO) in 1976, the population-based Saarland Cancer Registry (about 1.1 million residents) gradually used this classification to code NMSC reports. Due to the large number of NMSC reports and the limited number of registry staff, the majority of NMSC reports until 1994 were either only coded by the International Classification of Diseases, 9th revision (WHO, 1976), coded as unspecified skin cancer according to ICD-O (M800, M997), or coded by a histology variable that indicates whether the NMSC was a BCC, SCC or other skin cancer.

For the period 1995–1999, we checked all NMSC reports that either showed a mismatch between the histology variable and ICDO code, were coded as histologies that are usually not observed in the skin (e.g. leiomyosarcoma) or that were coded as lesions that do not belong to the group of NMSC (e.g. skin melanoma). Of the 6831 reports during this period, about 133 (2%) were checked. Seven of the 133 cancers were reclassified as other cancers or benign tumours, six out of 55 SCCs of the 133 reports were reclassified as BCCs and two out of 44 BCC reports were reclassified as SCCs.

After the data cleaning of the reports, we classified all reports according to the recommendations of the European Network of Cancer Registries (2000) on the classification of NMSCs based on the ICD-O third edition (BCC: M809–M811; SCC: M805–M808, M812–M813; and other or unspecified tumours). NMSC reports with missing ICD-O code were classified according to the histology variable of the cancer registry if this variable was nonmissing.

For the first occurrence of BCC and SCC, we restricted the calculation of incidence rates to the period 1995–1999 because before 1995, the annual proportion of unspecified histology codes was too large (>10%). Basal cell carcinoma and SCC counted only if the patient had no prior registered cancer with the same histologic diagnosis. Patients with a report both of BCC and SCC during the registration period were counted as cases within the BCC and SCC analysis.

We calculated site-specific incidence rates based on the fourth digit of the ICD-9 code (ICD-9: 173.0 skin of the lip, 173.1 eyelid, 173.2 external ear, 173.3 skin of other and unspecified parts of face, 173.4 skin of the scalp and neck, 173.5 skin of the trunk, 173.6 skin of the upper limb and shoulder, 173.7 skin of the lower limb and hip, 173.8 overlapping lesions of skin, 173.9 skin, not other specified).

We calculated sex-specific age-standardised incidence rates for BCC and SCC using the World Standard Population (Parkin *et al*, 1992) for the period 1995–1999. We also calculated age-specific incidence rates. For the study of the anatomical distribution of BCC and SCC, we calculated relative site-specific age-standardised incidence rates per unit area of the skin (RSA) for the period 1995–1999. We therefore divided the site-specific age-standardised incidence rates by the proportion of surface area of the corresponding body sites based on the estimates of the proportional surface area from Elwood and Gallagher (1998): face 2.3%, ear 0.5%, scalp and neck 6.1%, trunk 32%, arms and hands 16.5%, legs 40%. We estimated the proportional surface area of the lips (0.1%) and the eye lids (0.5%). We performed all analyses with SAS (SAS Inst., 1999).

Tables 1

**Table 1:** Age-specific, crude and age-standardised incidence rates of BCC and SCC among men in the Federal State of Saarland, 1995–1999

	**BCC**			**SCC**		
**Age (years)**	***N***	**Rate**	**s.e.**	***N***	**Rate**	**s.e.**
30–34	12	5.2	1.49	0	0.0	0.00
35–39	22	9.5	2.02	5	2.2	0.96
40–44	47	22.2	3.24	5	2.4	1.06
45–49	82	43.3	4.78	9	4.8	1.58
50–54	110	73.6	7.02	7	4.7	1.77
55–59	198	104.6	7.44	30	15.9	2.89
60–64	281	165.3	9.86	50	29.4	4.16
65–69	335	244.1	13.34	66	48.1	5.92
70–74	348	344.9	18.49	119	117.9	10.81
75–79	259	453.2	28.16	100	175.0	17.50
80–84	137	455.2	38.89	90	299.1	31.52
85+	116	565.6	52.51	62	302.3	38.39

*All ages*						
Crude	1960	74.9	1.69	543	20.8	0.89
WSR	1960	43.7	1.17	543	11.2	0.49

Rate: cases per 100 000; s.e.=standard error of the rate; crude=crude rate; WSR=age-standardised rate (World Standard Population); BCC=basal cell carcinoma; SCC=squamous cell carcinoma.

and 2

**Table 2:** Age-specific, crude and age-standardised incidence rates of BCC and SCC among women in the Federal State of Saarland, 1995–1999

	**BCC**			**SCC**		
**Age (years)**	***N***	**Rate**	**s.e.**	***N***	**Rate**	**s.e.**
30–34	26	11.7	2.30	0	0.0	0.00
35–39	34	15.4	2.64	0	0.0	0.00
40–44	53	26.3	3.61	4	2.0	0.99
45–49	79	43.8	4.93	1	0.6	0.56
50–54	88	59.8	6.38	5	3.4	1.52
55–59	178	91.2	6.83	17	8.7	2.11
60–64	224	123.9	8.28	24	13.3	2.71
65–69	243	153.0	9.81	23	14.5	3.02
70–74	287	179.1	10.57	50	31.2	4.41
75–79	305	267.8	15.34	72	63.2	7.45
80–84	253	331.5	20.84	83	108.7	11.94
85+	199	274.2	19.43	140	192.9	16.30

*All ages*						
Crude	1977	71.1	1.60	420	15.1	0.74
WSR	1977	31.7	1.06	420	4.4	0.31

Rate=cases per 100 000; s.e.=standard error of the rate; crude=crude rate; WSR=age-standardised rate (World Standard Population); BCC=basal cell carcinoma; SCC=squamous cell carcinoma.

present age-specific, crude and age-standardised incidence rates of first BCC and SCC among men and women from 1995 to 1999. Men had a 38% (95% CI: 27–50%) higher age-standardised incidence rate of BCC and a 55% (95% CI: 18–98%) higher age-standardised incidence of SCC than women. The median age at which a first BCC occurred was 68 years in men and 71 years in women, while that for first SCC was 74 years in men and 80 years in women.

The site-specific analyses of the age-standardised incidence rates of first BCCs and SCCs are presented in Tables 3

**Table 3:** Site-specific incidence rate ratios of first primary basal carcinoma in the Federal State of Saarland, 1995–1999

		**Males**				**Females**				**Sex ratio**	
**BCC**		***N***	**WSR**	**s.e.**	**RSA**	***N***	**WSR**	**s.e.**	**RSA**	**Male : female**	**95% CI**
Overall		1960	43.7	1.17		1977	31.7	1.06		1.38	1.27–1.50
	Surface (%)										
Lip (173.0)	0.1	26	0.56	0.13	5.60	41	0.57	0.13	5.70	0.98	0.52–1.86
Eye lid (173.1)	0.3	137	3.06	0.31	10.20	174	2.82	0.32	9.40	1.09	0.81–1.46
Ear (173.2)	0.5	113	2.42	0.25	4.84	32	1.15	0.20	2.30	2.10	1.46–3.04
Face (173.3)	2.3	900	20.0	0.78	8.70	961	14.5	0.69	6.30	1.38	1.22–1.55
Scalp/neck (173.4)	6.1	170	3.8	0.34	0.62	160	2.3	0.28	0.38	1.65	1.24–2.21
Trunk (173.5)	32.0	256	5.8	0.45	0.18	234	4.9	0.47	0.15	1.18	0.93–1.50
Arms (173.6)	16.5	117	2.8	0.32	0.17	78	1.3	0.22	0.08	2.15	1.46–3.18
Legs (173.7)	40.0	57	1.2	0.19	0.03	91	1.6	0.24	0.04	0.75	0.49–1.15
Overlapping (173.8)		1				1					
Unspecified (173.9)		183	4.1	0.36		205	3.3	0.34		1.24	0.95–1.62

WSR=age-standardised rate (World Standard Population), cases per 100 000; s.e.=standard error of the rate; RSA=age-standardised incidence rate divided by the proportion of surface area of the body site; BCC=basal cell carcinoma.

and 4

**Table 4:** Site-specific incidence rate ratios of first primary SCC in the Federal State of Saarland, 1995–1999

		**Males**				**Females**				**Sex ratio**	
**SCC**		***N***	**WSR**	**s.e.**	**RSA**	***N***	**WSR**	**s.e.**	**RSA**	**Male : female**	**95% CI**
Overall		543	11.2	0.49		420	4.4	0.31		2.55	2.18–2.98
	Surface (%)										
Lip (173.0)	0.1	25	0.53	0.13	5.30	5	0.10	0.07	1.00	5.30	1.73–16.3
Eye lid (173.1)	0.3	6	0.12	0.05	0.40	9	0.11	0.05	0.37	1.09	0.33–3.64
Ear (173.2)	0.5	95	1.95	0.19	3.90	17	0.13	0.03	0.26	15.0	8.56–26.3
Face (173.3)	2.3	163	3.36	0.27	1.46	203	2.13	0.21	0.93	1.58	1.23–2.02
Scalp/neck (173.4)	6.1	77	1.52	0.15	0.25	15	0.20	0.08	0.03	7.60	4.55–12.7
Trunk (173.5)	32.0	25	0.55	0.13	0.02	20	0.24	0.08	0.01	2.29	1.03–5.10
Arms (173.6)	16.5	64	1.34	0.18	0.08	70	0.67	0.11	0.04	2.00	1.30–3.07
Legs (173.7)	40.0	28	0.57	0.12	0.01	23	0.32	0.10	0.01	1.78	0.88–3.61
Overlapping (173.8)		0	—	—		1	—	—			
Unspecified (173.9)		60	1.21	0.16		57	0.55	0.10		2.20	1.41–3.42

WSR=age-standardised rate (World Standard Population), cases per 100 000; s.e.=standard error of the rate; RSA=age-standardised incidence rate divided by the proportion of surface area of the body site; SCC=squamous cell carcinoma.

. The risks of BCCs at the ears and the arms are higher for men than women. The risks of SCCs of the ears and scalp or neck are 15- and 7.6-fold higher among men than women. Although based on small numbers, the risk of SCCs at the lips appears to be 5.3-fold higher among men than women.

The comparison of the incidence rates by anatomic site is complicated because the anatomic sites have different surface areas. The site-specific age-standardised incidence rates for first BCCs and SCCs per unit area of the skin (RSA) correct for the different sizes of the surface areas. The comparison of the RSAs reveals that the risk of BCCs is highest at the eye lids, face and lips among both men and women. The risk of SCCs is highest at the lips, ears and face among men and lips, face and eye lids among women.

Nonmelanoma skin cancer results in relevant morbidity and mortality. This article provides population-based incidence estimates of BCC and SCC in West Germany. The incidence estimates of first occurrence are well comparable with the rates in other countries, including Denmark 1978–1982 (Osterlind *et al*, 1988), Finland 1991–1995 (Hannuksela-Svahn *et al*, 1999) and the Netherlands 1988 (Coebergh *et al*, 1991), but less than half of the rates in Australia (Buettner and Raasch, 1998).

Due to the large number of NMSC that were not coded according to the ICD-O before 1995 in the Saarland Cancer Registry, we were not able to analyse incidence time trends separately for BCC and SCC over a long period. The rising incidence of NMSC in the United States during the 1970s mainly affected BCC (Fears and Scotto, 1982). The age-adjusted incidence rate of SCCs in Arizona, USA, declined from 1985 to 1996 (Harris *et al*, 2001). Gallagher *et al* (1990) found similar incidence increases of BCC and SCC in British Columbia, Canada, from 1973 to 1987. These increases were most pronounced in the head and neck area. Levi *et al* (2001) observed an incidence increase of SCC and BCC in the Swiss Canton of Neuchatel from 1976 to 1998.

Body-site-specific incidence rates of both BCC and SCC showed wide variation with a clear preference for sun-exposed sites. The predilection of BCCs and SCCs for the head has been described previously (Pearl and Scott, 1986; Osterlind *et al*, 1988; Gallagher *et al*, 1990; Chuang *et al*, 1990a, 1990b; Coebergh *et al*, 1991; Serrano *et al*, 1991; Buettner and Raasch, 1998; Harris *et al*, 2001). The higher risk of NMSC of the ear, scalp and neck in men than women may be explained by the hairstyle among men, which less often covers these sites than among women. The excess risk of NMSC at the ears among men, which was also observed in Finland and the Netherlands (Coebergh *et al*, 1991; Hannuksela-Svahn *et al*, 1999), was mainly due to an excess risk of SCCs of the ears in our data. Smoking increases the risk of SCCs of the lip (Scotto *et al*, 1996; De Hertog *et al*, 2001) and may explain the higher risk of SCCs of the lips among men than women in our and other analyses (Coebergh *et al*, 1991; Buettner and Raasch, 1998). The risk of BCC of the legs among women is higher than among men, which has been also observed for skin melanoma and may correspond to the larger amount of sunlight exposure at the legs among women than men (Gallagher *et al*, 1990; Stang *et al*, 2003).

The public health problem of NMSC is several-fold. Incident cases of NMSC appear to be alarmingly common and increasing in frequency, even in young adulthood. They cause a considerable burden of morbidity, particularly deformity, as well as expense. Although death among NMSC cases is the exception rather than the rule, it also presents a considerable burden.

A limitation of our study results is the potential underestimation of the incidence rates due to incomplete registration of NMSCs that has been found by other cancer registries (Lucke *et al*, 1997; Hannuksela-Svahn *et al*, 1999; Maudsley and Williams, 1999). Unlike most other epithelial cancers, a large proportion of NMSC are easily treated by physicians (e.g. by cryotherapy, currettage, diathermy or excision) without histological confirmation of the diagnosis (Green, 1992) hampering the population-based registration of these tumours via pathology departments by the cancer registry. In addition, a variable proportion of people with skin cancer never present for medical treatment because of lack of symptoms or low levels of medical or public interest and such cases will escape conventional means of detection altogether. On the other hand, new cases of skin cancers, especially SCCs, may not be recognised or correctly diagnosed on presentation to doctors.
